# Multimodal Cardiac Imaging in Systemic Lupus Erythematosus: From Clinical Suspicion to Diagnosis in Clinical Practice

**DOI:** 10.3390/diagnostics16070988

**Published:** 2026-03-25

**Authors:** Mariagrazia Piscione, Barbara Pala, Francesco Cribari, Serena De Mitri, Giada La Placa, Dario Gaudio, Paola Gualtieri, Laura Di Renzo

**Affiliations:** 1 Fondazione Policlinico Campus Bio-Medico, University of Rome, Via Alvaro del Portillo 200, 00128 Rome, Italy; dario.gaudio@unicampus.it,; 2 PhD School of Applied Medical-Surgical Sciences, Tor Vergata University of Rome, Via Montpellier 1, 00133 Rome, Italy; laplacagiada@gmail.com; 3 UOC Cardiologia Ospedale, Istituto Dermopatico dell’Immacolata (IDI-IRCCS), 00167 Rome, Italy; fracrib93@gmail.com (F.C.); serenademitri3@gmail.com (S.D.M.); 4 Section of Food Science, Clinical Nutrition and Pharmaceutical Sciences, Department of Biomedicine and Prevention, Tor Vergata University of Rome, Via Montpellier 1, 00133 Rome, Italy; paola.gualtieri@uniroma2.it (P.G.); laura.di.renzo@uniroma2.it (L.D.R.)

**Keywords:** systemic lupus erythematosus, multimodal imaging, speckle tracking echocardiography, cardiac computed tomography, cardiac magnetic resonance

## Abstract

**Background:** Systemic lupus erythematosus (SLE) is a chronic autoimmune disease characterized by immune dysregulation and systemic inflammation, with the cardiovascular (CV) system representing a major yet frequently under-recognized target. Cardiac involvement spans from subclinical myocardial inflammation to overt pericardial disease, myocarditis, valvular abnormalities, coronary microvascular dysfunction, and accelerated atherosclerosis. Given that CV disease remains a leading cause of morbidity and mortality in SLE, early detection of silent cardiac injury is crucial. **Aim:** This review aims to provide a comprehensive and clinically oriented overview of CV involvement in SLE, focusing on the role of multimodal cardiac imaging in the detection, characterization, and risk stratification of cardiac abnormalities, as well as its potential implications for clinical management and preventive strategies. **Methods:** This narrative review is based on a structured, non-systematic search of PubMed (2013–2026), combining the term “systemic lupus erythematosus” with imaging-related keywords including “transthoracic echocardiography,” “cardiac magnetic resonance,” and “cardiac computed tomography.” English-language studies in adult populations were screened and selected according to clinical relevance, methodological robustness, and contribution to understanding SLE-related cardiac involvement. **Discussion:** Multimodal cardiac imaging plays a central role in the evaluation of SLE-related cardiac disease. Transthoracic echocardiography (TTE) represents the first-line modality for the assessment of ventricular function, pericardial disease, and valvular abnormalities, while deformation imaging enables the detection of subtle myocardial dysfunction. Cardiac magnetic resonance (CMR) provides comprehensive tissue characterization, allowing differentiation between active inflammation and chronic fibrosis. Cardiac computed tomography (cCT) identifies subclinical coronary atherosclerosis and high-risk plaque features, whereas nuclear imaging techniques offer insight into inflammatory activity and microvascular dysfunction. **Conclusions:** An integrated, imaging-based approach enables early diagnosis, refined CV risk stratification, longitudinal monitoring, and personalized therapeutic strategies. Multimodal imaging thus represents a key pillar of precision medicine in lupus-associated CV disease.

## 1. Introduction

Systemic lupus erythematosus (SLE) is an autoimmune disease characterized by systemic inflammation, aberrant immune activation and heterogeneous organ involvement [1].

This disease is driven by a complex interplay of immune system dysregulation, autoantibody generation, and persistent systemic inflammation [1]. The production of antinuclear and other pathogenic autoantibodies promotes the formation and deposition of immune complexes within various tissues, triggering complement activation, endothelial dysfunction, and inflammatory cascades that lead to progressive organ damage. In addition, antiphospholipid (APS) antibodies may contribute to a prothrombotic state, further exacerbating vascular injury and microvascular dysfunction [1].

Among affected organs, the cardiovascular (CV) system represents a major determinant of long-term prognosis, accounting for morbidity and premature mortality [1]. Indeed, CV events are more frequent in patients with SLE compared with age- and sex-matched general populations [2]. Cardiac involvement in SLE is multifaceted and may include pericardial disease, immune-mediated myocarditis, valvular abnormalities, coronary microvascular dysfunction, and accelerated atherosclerosis [3]. Many of these manifestations remain clinically silent for prolonged periods, delaying diagnosis and therapeutic intervention [3].

Traditional clinical evaluation and circulating biomarkers often lack sensitivity for early cardiac involvement [4]. As a result, cardiac disease in SLE is frequently detected only after irreversible myocardial damage has occurred [4]. In this context, multimodal cardiac imaging has emerged as a cornerstone for the comprehensive evaluation of SLE-related CV disease, offering unique insights into cardiac structure, function, tissue composition, and vascular integrity [5].

Beyond its diagnostic value, cardiac imaging increasingly influences clinical decision-making by refining CV risk stratification, guiding cardioprotective therapies, and enabling longitudinal monitoring of disease activity and treatment response [5].

This review is structured in two complementary sections. The first part provides a comprehensive overview of SLE-related cardiac manifestations, with particular emphasis on the role of imaging in their detection, characterization, and pathophysiological interpretation. The second part focuses on contemporary evidence derived from recent clinical studies, highlighting how different imaging modalities have been applied in SLE populations and identifying the main research directions currently shaping the field.

In addition, this review aims to provide a clinically oriented and integrative perspective that goes beyond narrative summaries by linking multimodal imaging findings with underlying pathophysiological mechanisms and their potential implications for CV risk stratification and management. Furthermore, the present work emphasizes the complementary role of TTE, CMR, cCT, and nuclear imaging within a unified framework, considering emerging concepts such as subclinical myocardial involvement, microvascular dysfunction, and imaging-guided preventive strategies, thereby offering a translational and practice-oriented approach.

## 2. Literature Search and Review

**Figure 1:**
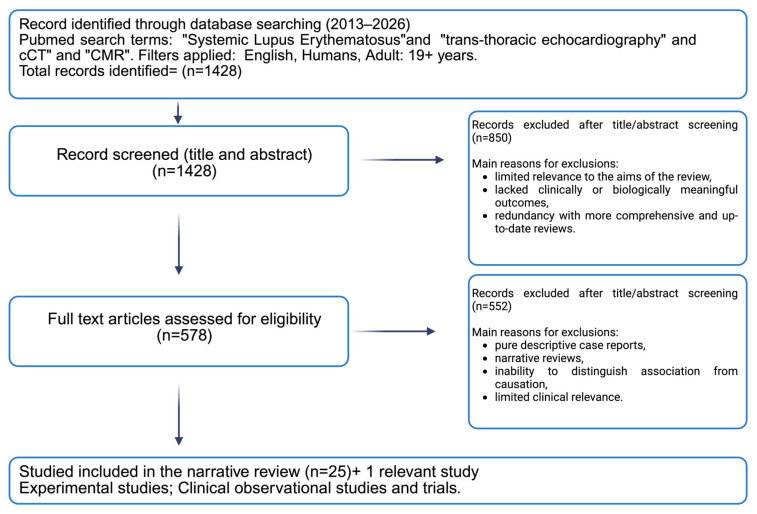
Literature search and study selection.

This narrative review is based on a structured, non-systematic search of PubMed (2013–2026) (Figure 1). The search strategy combined the term “systemic lupus erythematosus” with imaging-related keywords using Boolean operators (AND/OR), including “transthoracic echocardiography,” “cardiac magnetic resonance,” “cardiac computed tomography,” “coronary CT angiography,” and “nuclear imaging.” Relevant Medical Subject Headings (MeSH) terms were also considered where applicable.

Only English-language studies involving adult populations were included. Studies were selected based on clinical relevance, recency, and methodological robustness. Priority was given to original research articles, prospective studies, and studies employing advanced imaging techniques, while review articles were used to support contextual interpretation.

Methodological robustness was qualitatively assessed based on study design, sample size, imaging methodology, and consistency of findings across studies.

## 3. Pathophysiology of Cardiac Involvement in SLE: Implications for Imaging

### 3.1. Systemic Autoimmunity in SLE

The immune system is a complex of cells, organs, and molecular mediators designed to recognize and eliminate pathogens while preserving self-tolerance [6]. Two arms—innate and adaptive immunity—coordinate inflammatory responses, tissue repair, and host defence [7]. When immune regulation fails, the system loses the ability to discriminate between self- and non-self-antigens, resulting in an immune response directed against host tissues [7].

This breakdown of immune tolerance leads to persistent immune activation, even in the absence of infection, with alternating phases of heightened inflammatory activity and relative quiescence [8]. Such dysregulated immunity underlies autoimmune diseases, in which self-reactive immune responses cause progressive tissue and organ damage [8]. Mechanistically, molecular mimicry between microbial antigens and host structures may initiate autoimmunity, while bystander activation further amplifies immune responses by recruiting additional immune pathways [9]. SLE represents a systemic autoimmune disease characterized by a general degree of inflammation, and a relapsing–remitting clinical course. SLE can virtually affect any organ system, most commonly involving the joints, skin, kidneys, lungs, and central nervous system [9]. The marked heterogeneity of immune responses and clinical manifestations complicates diagnosis and contributes to delays in disease recognition [10].

### 3.2. Epidemiology and Clinical Heterogeneity of SLE

SLE affects women of childbearing age, with a peak incidence between 15 and 40 years. The female-to-male ratio ranges from approximately 6:1 to 10:1, highlighting a strong sex-related predisposition [11]. In the United States, the estimated prevalence ranges from 14.6 to 50.8 cases per 100,000 individuals. Hormonal factors appear to contribute to disease susceptibility, as exposure to exogenous oestrogens—such as oral contraceptives or hormone replacement therapy—has been associated with an increased risk of SLE development [11]. The multisystem nature of SLE results in a wide spectrum of clinical presentations. Early or recurrent disease flares commonly manifest with non-specific symptoms such as fatigue, fever, and weight changes [12]. Musculoskeletal involvement is often the initial presentation, with arthritis and arthralgia affecting predominantly small joints of the hands and wrists. Unlike inflammatory arthritis in other rheumatic diseases, joint pain in SLE may be disproportionate to objective observable swelling [12]. Cutaneous involvement is also frequent and may be classified as acute, subacute, or chronic based on timing and morphology [12]. Classic manifestations include malar rash, photosensitivity, discoid lesions with follicular plugging and scarring, and alopecia [13]. Renal involvement represents one of the most severe disease manifestations, with lupus nephritis (LN) affecting approximately 50% of Caucasian and up to 75% of African American patients [13]. Nearly all patients demonstrate some degree of renal impairment over the disease course, reflected by abnormalities in serum creatinine, hematuria, or pyuria [13]. Neurological manifestations can occur in most patients and include a broad spectrum of central, peripheral, and autonomic nervous system involvement [13]. Symptoms comprise headaches, mood disorders, cognitive dysfunction, seizures, cerebrovascular events, transverse myelitis, and peripheral neuropathies, contributing to significant diagnostic and therapeutic complexity [14].

### 3.3. Cardiovascular Involvement and Vascular Inflammation in SLE

CV and pulmonary involvement are closely intertwined in SLE and represent a major determinant of long-term prognosis [15]. Immune-mediated vascular injury promotes endothelial dysfunction and accelerates atherosclerosis, markedly increasing CV risk even in young patients [15]. Consequently, chest pain, dyspnoea, and heart failure (HF) symptoms in SLE require careful evaluation [15].

Vascular inflammation predisposes patients to a broad range of CV manifestations, including myocarditis, coronary vasculitis, valvular disease, endocarditis, coronary microvascular dysfunction, and premature coronary artery disease [15]. Despite increasing recognition of the burden of CV diseases in SLE, the mechanisms linking systemic autoimmunity to CV injury remain incompletely understood, underscoring the need for improved diagnostic strategies and preventive approaches [15].

### 3.4. Pathophysiology and Predisposing Factors

The pathogenesis of SLE arises from the interplay of genetic susceptibility, environmental triggers, hormonal influences, and immune dysregulation [16]. Loss of immune tolerance results in activation of autoreactive B and T lymphocytes, leading to excessive autoantibody production and immune complex formation [16].

Dysregulated B-cell tolerance is a hallmark of SLE and results in the generation of a broad array of autoantibodies targeting nuclear, cytoplasmic, and cell surface antigens [16]. Antinuclear antibodies are present in over 95% of patients, while anti-double-stranded DNA antibodies are closely associated with disease activity and organ damage [17]. Immune complexes derived from apoptotic cells amplify inflammation and contribute to tissue injury through complement activation and Fc receptor engagement [17].

Innate immune cells, including dendritic cells and neutrophils, further propagate autoimmunity [17]. Neutrophil extracellular trap (NET) formation enhances antigen exposure and perpetuates immune activation [17]. Abnormal cytokine profiles, particularly involving type I interferons, also play a central role in sustaining chronic inflammation [17]. Genetic factors contribute significantly to disease susceptibility, as demonstrated by high concordance rates among monozygotic twins [18]. Approximately 150 genetic loci have been implicated, including genes involved in antigen presentation, immune signaling, and interferon pathways [18]. Epigenetic modifications, such as DNA hypomethylation, further modulate disease risk. As already said above, hormonal influences are evident in the pronounced female predominance of SLE, with oestrogen enhancing immune activation and altered sex hormone profiles observed in affected individuals [18].

### 3.5. Low-Grade Cardiovascular Inflammation as a Link Between Systemic Autoimmunity and Cardiovascular Risk in SLE

In SLE, chronic immune dysregulation does not translate into continuous high-grade inflammation but rather into a state of persistent, low-grade inflammatory activation that exerts cumulative harmful effects on the CV system [19]. Even during periods of clinical remission, subclinical immune activation persists, characterized by circulating immune complexes and sustained cytokine signaling [19]. This chronic inflammatory milieu promotes progressive vascular and myocardial injury, often in the absence of overt CV symptoms [19]. At the vascular level, immune-mediated endothelial dysfunction represents an early and central mechanism linking systemic autoimmunity to CV disease [19]. Pro-inflammatory cytokines, autoantibodies, and activated immune cells impair endothelial nitric oxide bioavailability, increase oxidative stress, and disturb vascular homeostasis [19]. These alterations favour coronary microvascular dysfunction and accelerate atherosclerotic processes, even in young patients with few traditional CV risk factors [20]. Within the myocardium, low-grade inflammation contributes to subtle alterations in myocardial structure and mechanics [20]. Repeated episodes of immune-mediated injury, insufficient to cause myocarditis, may nonetheless result in diffuse interstitial fibrosis, impaired myocardial relaxation, diastolic dysfunction or early systolic dysfunction [20]. Over time, these changes create a substrate for HF, arrhythmias, and adverse CV outcomes [20]. This process highlights the disconnect between clinical quiescence and ongoing myocardial remodeling in SLE [20]. Importantly, recognizing low-grade CV inflammation as a key pathophysiological driver provides a conceptual framework for early detection and prevention.

## 4. Spectrum of Cardiovascular Diseases in SLE

Cardiac involvement in SLE may affect all structural components of the heart, including the pericardium, myocardium, endocardium, coronary vasculature, and the cardiac conduction system, often coexisting within the same patient [21].

### 4.1. Pericardial Disease in SLE

#### 4.1.1. Epidemiology, Pathogenesis and Clinical Presentation

Pericardial disease is the most encountered form of cardiac involvement in SLE and reflects the vulnerability of serosal tissues to immune-mediated damage [22]. Pericardial involvement may arise at any stage of the disease and frequently remains unnoticed due to subtle or asymptomatic presentation [22].

The underlying mechanisms of SLE-related pericardial disease involve immune complex deposition within the pericardial layers, activation of the complement cascade, and sustained local cytokine release [23]. These processes lead to inflammatory infiltration, increased capillary permeability, and accumulation of pericardial fluid [23,24].

From a clinical standpoint, pericardial involvement spans a wide spectrum, ranging from incidental findings such as minimal effusions or pericardial thickening to acute pericarditis with chest pain and inflammatory signs [25]. Pericarditis in patients with SLE typically presents the classic signs and symptoms of acute pericarditis [25]. Patients most commonly report pleuritic precordial or substernal chest pain with positional variation, often relieved by sitting upright [25]. Associated symptoms may include dyspnoea, fever, tachycardia, and muffled heart sounds [25]. A pericardial friction rub, when present, is supportive of the diagnosis, although its absence does not exclude pericardial inflammation [26].

Recurrence following an initial episode of pericarditis is relatively common, occurring in approximately 15–30% of cases [26]. By contrast, cardiac tamponade and constrictive pericarditis are uncommon in SLE, affecting approximately 2% of patients [26].

**Figure 2:**
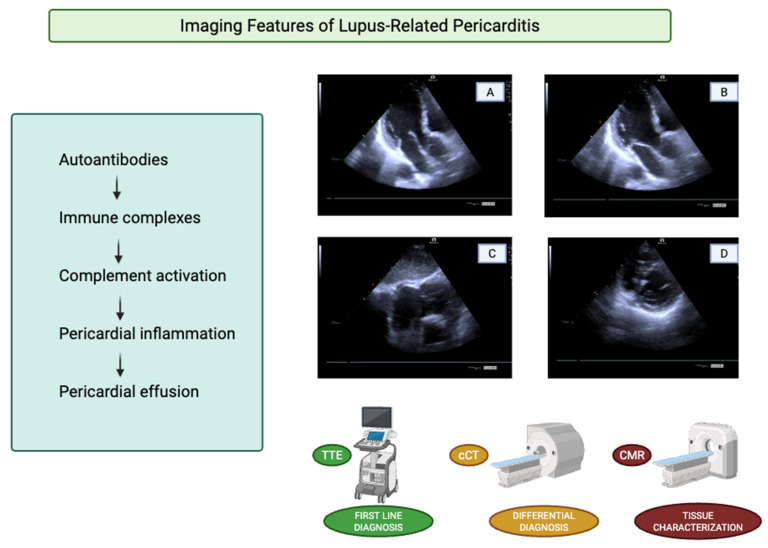
**Imaging features of lupus-related pericarditis.** (**A**,**B**) Apical three-chamber view demonstrating increased pericardial echogenicity along the postero-lateral wall in diastole and systole respectively. (**C**) Subcostal view showing a bright, thickened pericardial line and a mild pericardial effusion anteriorly. (**D**) Parasternal short-axis view illustrating circumferential pericardial hyperechogenicity, supporting the diagnosis of non-effusive lupus-related pericarditis.

Overall, pericardial disease in SLE generally follows a benign course but requires careful clinical and imaging surveillance due to the risk of recurrence and the potential development, albeit rare, of hemodynamically significant complications (Figure 2) [27].

#### 4.1.2. Imaging Assessment of Pericardial Involvement

**Figure 3:**
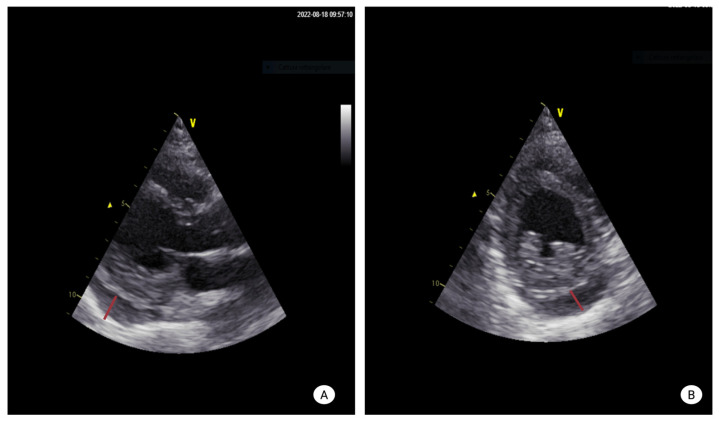
**Imaging features of severe pericardial effusion in a 40-years- old SLE.** (**A**) Transthoracic echocardiography, parasternal long-axis view, showing a large pericardial effusion predominantly along the posterolateral wall. (**B**) Parasternal short-axis view confirming the presence of a circumferential pericardial effusion, more evident along the lateral wall.

**Figure 4:**
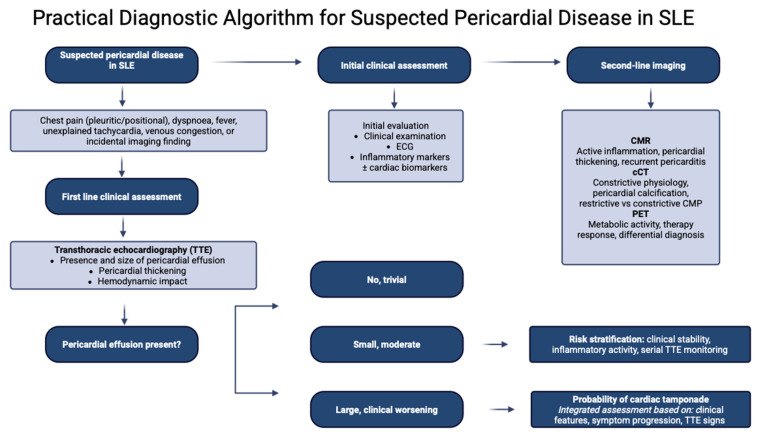
This figure summarizes a practical diagnostic algorithm for the evaluation of suspected pericardial disease in patients with SLE, integrating clinical assessment and multimodal imaging.

Although several imaging modalities are available for the evaluation of pericardial disease, TTE is universally regarded as the first-line imaging technique [28]. Doppler TTE enables assessment of pericardial thickness, characterization of pericardial fluid, and evaluation of the physiological and hemodynamic consequences of an effusion [28]. Under normal conditions, a small amount of pericardial fluid may be visualized only during systole; as fluid accumulates, it becomes evident throughout the entire cardiac cycle [28]. The detection of a circumferential effusion from multiple two-dimensional imaging windows improves the accuracy of effusion size estimation [28]. Pericardial effusions are commonly classified according to the end-diastolic separation between the parietal and visceral pericardium as trivial, small, moderate, or large [29]. TTE signs suggestive of tamponade physiology include early diastolic right ventricular collapse, late diastolic right atrial collapse, respiratory variation in mitral inflow velocities, and inferior vena cava dilation with reduced respiratory collapse; however, these findings must always be interpreted in conjunction with clinical presentation [29]. As a matter of fact, recent European guidelines on pericardial diseases propose a diagnostic algorithm to estimate the probability of cardiac tamponade based on the integration of clinical, anamnestic findings and TTE features [29]. TTE may also assist in differentiating acute pericarditis from myocardial ischemia by excluding regional wall motion abnormalities, although transient abnormalities may rarely be observed in acute pericarditis [29]. In uncommon cases, autoimmune diseases may be complicated by constrictive pericarditis, which should be suspected in the presence of interventricular septal bounce or respiro-phasic septal shift [29] (Figure 2, Figure 3 and Figure 4).

cCT provides high-resolution, motion-independent visualization of the pericardium and allows multiplanar image reconstruction for detailed anatomical assessment [30]. The pericardium appears as a thin, hyperattenuating linear structure, readily distinguishable from surrounding epicardial fat on both contrast-enhanced and non-contrast studies [30]. Contrast enhancement of thickened pericardial layers supports the diagnosis of active inflammation, while the combination of non-calcified pericardial thickening and effusion is suggestive of acute pericarditis [31]. cCT demonstrates high specificity for detecting pericardial thickening and is particularly valuable in differentiating constrictive pericarditis from restrictive cardiomyopathy, given the marked increase in pericardial thickness typically observed in constriction [31]. Furthermore, cCT represents the reference imaging modality for identifying pericardial calcifications, which are present in approximately half of patients with constrictive pericarditis and are most frequently located along the atrioventricular grooves and basal cardiac regions [31].

CMR offers comprehensive assessment of pericardial disease by combining detailed tissue characterization with functional and hemodynamic evaluation, particularly in cases where TTE findings are inconclusive [32]. CMR employs multiple sequences, including T1- and T2-weighted imaging, cine acquisitions, and late gadolinium enhancement (LGE), to identify pericardial thickening, oedema, and inflammation [32]. The absence of pericardial thickening or enhancement on CMR carries a high negative predictive value in patients with recurrent pericarditis [32]. Serial CMR examinations may therefore be used to monitor inflammatory activity and guide therapeutic decisions [33]. LGE of the pericardium is a sensitive marker of active inflammation and is frequently observed in both chronic recurrent pericarditis and constrictive forms [33]. Advanced techniques such as CMR tagging may further assist in identifying fibrotic adhesions between pericardial layers and assessing concomitant myocardial involvement [33].

Positron emission tomography (PET) may serve as an adjunctive imaging modality by providing metabolic information on pericardial inflammation. Unlike malignant processes, which typically demonstrate intense focal uptake of 18F-fluorodeoxyglucose, autoimmune or idiopathic pericarditis generally exhibits mild-to-moderate uptake or may even appear metabolically inactive [34]. PET imaging can therefore be useful in selected cases to assess inflammatory activity and to monitor response to therapy over time [34,35].

### 4.2. Myocardial Disease in SLE

#### 4.2.1. Epidemiology, Pathogenesis and Clinical Presentation

Clinical studies have reported myocarditis in approximately 9% of patients with SLE, whereas earlier post-mortem series described a markedly higher prevalence, exceeding 50%, highlighting the frequent occurrence of subclinical myocardial involvement [36]. The reduction in myocarditis prevalence observed in the last twenty years in autopsy studies is likely related to the widespread introduction of corticosteroid therapy, which has significantly modified the natural history of cardiac involvement in SLE [37].

About the physiopathology, myocardial damage in SLE is largely driven by immune-mediated mechanisms [37]. Immunofluorescence analyses have demonstrated granular deposition of immune complexes and complement components within the walls and perivascular regions of myocardial vessels, with scattered immune deposits also observed along myocyte bundles [38]. Inflammatory infiltrates tend to display a focal distribution, whereas immune reactants are more diffusely present, supporting the concept that SLE- myocarditis primarily reflects an immune complex-mediated vascular process rather than direct primary injury of cardiomyocytes [38]. Complement activation and subsequent inflammatory cascades appear central to the development of myocardial dysfunction [39].

Several circulating autoantibodies—including anti-Sjögren’s-syndrome-related antigen A (anti-Ro/SSA), antiribonucleoprotein, antimyocardial, and antiphospholipid antibodies—have been implicated [40]. Some studies suggest a potential association between anti-Ro antibodies and both myocarditis and conduction abnormalities, whereas others have not confirmed this link [40]. Antiribonucleoprotein antibodies may identify a subset of patients prone to combined cardiac and myositis, suggesting immune targeting of striated muscle [40]. However, antimyocardial antibodies, while frequently detected, lack specificity for active myocardial inflammation [40].

Histopathological findings typically include interstitial oedema, focal inflammatory infiltrates composed of lymphocytes and plasma cells, fibrinoid changes in collagen, immune complex deposition in perivascular areas, myocyte necrosis, and vascular remodeling with intimal thickening and luminal narrowing [41]. Over time, resolution of inflammation may result in patchy myocardial fibrosis, even in the absence of significant coronary artery disease [41]. In rare cases, more aggressive forms such as haemorrhagic myocarditis have been reported [41]. Involvement of the cardiac conduction system may occur through inflammatory damage, scarring of the sinus and atrioventricular nodes, or injury to the bundle branches and nodal vasculature [41]. Progressive necrosis and fibrotic replacement may ultimately lead to chronic myocarditis and dilated cardiomyopathy [41].

SLE is most often clinically silent but may present with non-specific symptoms [42]. Physical findings may include jugular venous distension, resting tachycardia, gallop rhythms, new cardiac murmurs, cardiomegaly, and peripheral oedema [42]. According to Thomas et al., analysing a total of 29 patients, SLE- myocarditis represents a severe manifestation of the disease and may occur either as the initial presentation of the pathology or during follow-up, particularly in untreated patients [43]. Despite its potentially life-threatening nature, the long-term prognosis is generally favorable. In patients with milder forms of myocardial involvement, recovery of left ventricular ejection fraction (LVEF) is often observed even in the absence of cyclophosphamide therapy [43].

#### 4.2.2. Imaging Assessment in Myocarditis

TTE plays a key role in the non-invasive evaluation of suspected myocarditis by allowing assessment of the presence, distribution, and functional impact of myocardial inflammation [44]. Although it cannot definitively confirm the diagnosis, TTE may reveal global or regional wall motion abnormalities suggestive of inflammatory myocardial involvement [44]. Moreover, a retrospective study of 28 patients with SLE-myocarditis showed that speckle-tracking-derived global longitudinal strain (GLS) correlates with LVEF, detects subclinical myocardial impairment despite preserved LVEF [45]. Moreover, the introduction of CMR has had a pivotal role as a matter of fact, in a retrospective study comparing patients with suspected SLE- myocarditis before and after the introduction of CMR, the implementation of CMR has been associated with a significant reduction in the use of endomyocardial biopsy and a lower rate of immunosuppressive therapy, supporting the role of CMR as a non-invasive diagnostic tool that influences both diagnostic strategies and clinical management [46,47,48,49,50].

### 4.3. Valvular Heart Disease in SLE

#### 4.3.1. Epidemiology, Pathogenesis and Clinical Presentation

Valvular involvement is a well-recognized manifestation of SLE and reflects immune-mediated injury of the endocardial surface [51]. The major expression of SLE-related valvular disease is Libman–Sacks endocarditis, a form of nonbacterial thrombotic endocarditis characterized by sterile verrucous vegetations composed of immune complexes, fibrin, and platelet aggregates [52]. First described in 1924, this form of endocarditis is characterized by sterile vegetations that most commonly involve the mitral and aortic valves [52]. CV interest is a major contributor to morbidity in SLE, and improvements in survival have increasingly highlighted the clinical relevance of valvular disease [53]. Although valvular thickening and subclinical lesions are common—reported in up to 30–50% of autopsy series—clinically significant valvular dysfunction remains relatively uncommon [53]. Prospective TTE studies have demonstrated a prevalence of Libman–Sacks vegetations ranging from approximately 9–11%, with higher overall rates of valvular abnormalities when leaflet thickening is included [53]. Importantly, several investigations have shown a significant association between valvular lesions and antiphospholipid antibodies, particularly anticardiolipin antibodies, although this relationship has not been consistently confirmed across imaging modalities [54]. Long-term cohort studies suggest that while valvular abnormalities may progress over time, only a minority of patients develop severe regurgitation requiring surgical intervention, and high titers of IgG anticardiolipin antibodies appear to identify individuals at increased risk of severe dysfunction [54].

The pathogenesis of Libman–Sacks endocarditis remains incompletely understood but is believed to involve immune complex deposition and complement activation at the valvular surface, leading to endothelial injury [54]. Subsequent deposition of fibrin–platelet thrombi promotes leaflet thickening, fibrosis, and, eventually, verrucous vegetations [54]. Histopathological findings typically include fibrin deposits, neovascularization, hyaline changes, calcification, and variable mononuclear inflammatory infiltrates [54]. In patients with antiphospholipid syndrome—either primary or secondary to SLE—the prothrombotic milieu further amplifies thrombus formation on previously damaged valves, suggesting a synergistic interaction between immune-mediated injury and thrombosis [54,55].

Clinically, Libman–Sacks endocarditis is often asymptomatic but may lead to embolic complications, superimposed infective endocarditis, or progressive valvular regurgitation [56]. When symptomatic, its presentation may mimic infective endocarditis, making differential diagnosis challenging [56]. In this context, the modified Duke criteria remain useful for excluding bacterial infection [57]. Laboratory findings may aid differentiation: leukopenia during SLE flare contrasts with leukocytosis in infection, markedly elevated C-reactive protein levels favour infective endocarditis, and moderate-to-high APS antibody titres support a SLE-related aetiology [57]. Overall, Libman–Sacks endocarditis reflects a continuous pathogenic process beginning with immune-mediated endothelial injury, progressing to leaflet thickening, and culminating in vegetations and their potential thromboembolic consequences [58].

#### 4.3.2. Imaging Assessment in Valvular Heart Disease

TTE represents the primary imaging modality for the evaluation of valvular involvement in SLE and plays a central role in diagnosing Libman–Sacks endocarditis as well as in differentiating it from infective endocarditis and intracardiac tumors [58]. On TTE examination, Libman–Sacks vegetations typically appear as irregular, sessile masses measuring more than 2 mm in diameter, firmly attached to the valvular surface and lacking independent oscillatory motion [59]. Their borders are often ill-defined, and they are most commonly located on the atrial surface of the mitral valve or the ventricular surface of the aortic valve, with left-sided valves more frequently involved [60]. Although vegetations may occasionally arise at commissures, free margins, or annular regions, extension to the subvalvular apparatus—including chordae tendineae, papillary muscles, and adjacent mural endocardium—has been described, particularly in mitral involvement [61]. Valvular thickening represents the most frequent abnormality, while regurgitation is the predominant functional consequence, with mitral regurgitation being more common than aortic or tricuspid regurgitation [61]. Transesophageal echocardiography provides superior sensitivity compared with TTE and is particularly useful in detecting small vegetations or subtle leaflet abnormalities [61]. In contrast, vegetations in infective endocarditis are more commonly located along the line of leaflet closure, tend to be mobile with independent motion, and typically exhibit a more homogeneous echogenic appearance [62]. Careful evaluation of vegetation morphology, attachment site, mobility, and associated valvular dysfunction is therefore essential to establish the correct diagnosis and guide appropriate management [62].

### 4.4. Arrhythmias and Conduction Abnormalities in SLE

Conduction system involvement has been described, including atrioventricular block and bundle branch block, and may reflect inflammatory or ischemic injury to the specialized conduction tissue [63]. Arrhythmic manifestations in SLE are frequently described in the fetal or neonatal setting, particularly in association with transplacental autoantibody exposure, whereas in adults they are often secondary to underlying structural cardiac involvement such as myocarditis or fibrosis; for this reason, arrhythmias are addressed only briefly in this review [63].

### 4.5. Coronary Microvascular Dysfunction and Accelerated Atherosclerosis in SLE

CV disease is a major determinant of mortality in SLE [64]. Early descriptions report a bimodal mortality pattern, with deaths in the first year largely related to active disease and later mortality predominantly driven by CV complications [64]. Although advances in immunosuppressive therapy have improved overall survival, contemporary data indicate that CV diseases and infections remain the leading causes of death throughout the SLE course [65]. The risk of myocardial infarction is approximately ten-fold higher in SLE compared with the general population, even after adjustment for traditional risk factors, and is particularly striking in young women, in whom relative risk may exceed fifty folds compared with age-matched controls [66].

Despite this marked relative risk, the absolute annual number of CV events in individual cohorts remains modest, prompting growing interest in surrogate markers of subclinical vascular disease [67]. Carotid ultrasound studies consistently demonstrate increased plaque prevalence and faster progression in SLE, while coronary artery calcification is more frequent than in matched controls [68]. Functional imaging further reveals vascular dysfunction, including myocardial perfusion abnormalities and impaired endothelial function [68]. Importantly, vascular involvement is not limited to large vessels: reduced coronary flow reserve has been documented even in patients without obstructive coronary disease, suggesting concomitant microvascular dysfunction [69]. However, although these subclinical measures are predictive of events in the general population, robust longitudinal data linking most surrogate markers to hard CV outcomes in SLE remain limited, with abnormal myocardial perfusion being among the few consistently associated with future events [70].

From a pathophysiologic perspective, atherosclerosis is a chronic inflammatory process characterized by accumulation of apoptotic debris and oxidized low-density lipoproteins (OxLDL), along with infiltration of activated T lymphocytes and monocyte–macrophages producing pro-inflammatory cytokines such as interleukin-1, interleukin-6, and tumour necrosis factor-α [71]. Sustained inflammation promotes plaque instability and calcification [71]. The inflammatory hypothesis of atherosclerosis is supported by interventional evidence: inhibition of interleukin-1β with canakinumab reduced major CV events independently of lipid lowering, and colchicine has demonstrated benefit in secondary prevention [72].

In SLE, the link between immune dysregulation and vascular disease is particularly compelling. Both conditions share impaired clearance of apoptotic cells and increased OxLDL levels, fostering persistent immune activation within the vascular wall [73]. CV risk in SLE likely reflects the combined influence of traditional risk factors and disease-specific contributors, including chronic inflammation, cumulative organ damage, autoantibodies, altered lipid profiles, reduced levels of protective antibodies such as anti-phosphorylcholine, and genetic susceptibility [73]. While atherosclerosis is often considered “accelerated” in SLE, longitudinal studies have yielded heterogeneous results, with some cohorts showing no significant difference in intima–media thickness progression despite greater plaque burden at baseline [74]. These findings highlight the need for prospective studies capable of distinguishing plaque formation from intimal thickening and identifying high-risk plaque phenotypes [75]. Overall, current evidence supports the concept of a pro-inflammatory vascular environment in SLE that promotes both macrovascular and microvascular dysfunction, although the precise dynamics of plaque progression remain to be fully clarified [75].

The following sections provide an overview of the available literature for each imaging modality, highlighting the specific cardiac and vascular domains that have been most extensively investigated in patients with SLE.

## 5. Imaging Evaluation in Systemic Lupus Erythematosus

### 5.1. Trans-Thoracic Assessment in SLE Patients

As previously discussed, TTE has emerged as a cornerstone for the evaluation of both overt and subclinical cardiac involvement in SLE [76,77,78,79,80,81,82]. Conventional assessment of systolic function relies on LVEF; however, this parameter typically decreases only in advanced stages of myocardial disease and therefore lacks sensitivity for the detection of early contractile defects [83]. In recent years, speckle-tracking TTE has emerged as a more sensitive technique for identifying subtle myocardial impairment [83]. Left ventricle mechanical dispersion (LVMD) abnormalities, calculated as the standard deviation of the time-to-peak longitudinal strain for all the LV segments, is also frequently observed despite preserved LVEF [83]. As a matter of fact, Shahab et al. identify increased LVMD in patients with SLE, with complement protein C4 emerging as a predictor of LVMD, underscoring the link between inflammatory burden and myocardial involvement [83]. In line with this, Morello et al. demonstrate reduced GLS in nearly one-quarter of patients with SLE, associated with diastolic dysfunction, right ventricular impairment, prior pericardial involvement, and elevated inflammatory markers, supporting a shared immune-mediated pathophysiological substrate [76]. Lai et al. further confirm the prognostic role of myocardial deformation indices, showing that increased left ventricular mass, impaired diastolic indices, and abnormal strain parameters independently predicted CV events [78].

Moreover, left atrial (LA) strain has increasingly been recognized as a reliable noninvasive parameter for estimating left ventricular filling pressures [84]. Derived from speckle-tracking TTE, LA strain can be obtained on most contemporary ultrasound platforms and allows both real-time and offline analysis [85]. Its diagnostic performance in estimating LA pressure appears particularly robust in patients with reduced LVEF [85]. Beyond reservoir function alone, the ratio between LA reservoir strain and the transmitral E/e′ index can be used to calculate a non-invasive marker of LA stiffness [86]. This composite index has demonstrated superior accuracy compared with conventional TTE parameters in identifying patients with HF with preserved ejection fraction and in stratifying those at higher risk of hospitalization [86]. In a cohort study by Zhong et al. patients with SLE exhibited increased LA stiffness index and impaired reservoir function, particularly in those with LN, suggesting that LA strain may serve as an early marker of subclinical diastolic dysfunction [87]. Similarly, Dai et al. reported reduced LA reservoir and conduit function with compensatory augmentation of pump function, closely associated with worsening diastolic dysfunction and cumulative disease damage [82].

Right heart involvement in SLE-related pathogenesis is equally relevant, particularly in the presence of pulmonary hypertension (PH). Sun et al. [81] showed progressive right atrial remodeling with worsening PH severity, characterized by reduced passive and systolic strain parameters and compensatory increases in active emptying [81]. Finally, valvular and pericardial abnormalities remain prevalent even in subclinical stages, as reported by Mohamed et al. [80], further supporting the role of comprehensive TTE evaluation in routine clinical follow-up.

Collectively, these studies indicate that TTE—especially when combined with myocardial deformation analysis—enables early detection of subclinical systolic and diastolic dysfunction, atrial remodeling, right heart impairment, and vascular interaction in SLE, thereby offering both diagnostic and prognostic value (Table 1).

### 5.2. cCT Assessment in SLE Patients

**Figure 5:**
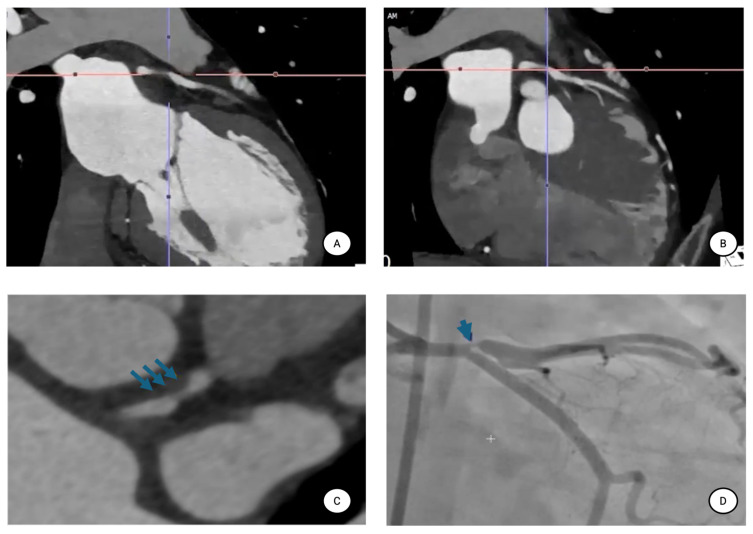
**Multimodal assessment of coronary artery disease in a patient with systemic lupus erythematosus.** This figure illustrates a representative case of a 53-year-old woman with SLE presenting with dyspnea on mild to moderate exertion. (**A**–**C**) CCTA with cross-sectional and multiplanar reconstructions demonstrates a markedly eccentric, non-calcified plaque in the left anterior descending artery (LAD), characterized by high-risk features including low attenuation and positive remodeling, resulting in severe luminal narrowing (CAD-RADS 4). (**D**) Invasive coronary angiography confirms the severity of the stenosis in the corresponding LAD segment (blue arrow). This case highlights the potential role of CCTA as a valuable non-invasive tool for the early detection and risk stratification of coronary artery disease in patients with SLE. Abbreviations: CAD-RADS: Coronary Artery Disease–Reporting and Data System; CCTA: coronary computed tomography angiography; LAD: left anterior descending artery; SLE: systemic lupus erythematosus.

CCT has significantly contributed to the understanding of premature atherosclerosis in SLE [44,88,89,90,91,92,93,94] (Figure 5). Early evidence by Yiu et al. demonstrates a markedly higher prevalence of coronary artery calcification (CAC) in patients with SLE compared with controls, involving not only the coronary arteries but also carotid arteries and the aorta, suggesting diffuse vascular interest [88].

Following studies confirm that coronary calcification develops earlier in SLE and is not fully explained by traditional CV risk factors [89]. In a case–control study by Khan et al., increased CAC burden is observed in SLE patients independently of both conventional and disease-specific risk factors, supporting the role of SLE-related inflammatory mechanisms in vascular calcification [89]. Similarly, Gartshteyn et al. show that non-contrast calcium scoring is useful for detecting early plaque burden, reinforcing its potential role in CV risk stratification [91]. More recently, Wu et al. highlight the strong negative predictive value of a negative calcium score in primary prevention settings [94].

Beyond calcification, cCT has revealed a high prevalence of non-obstructive plaques, even in asymptomatic patients [90]. Hermansen et al. report frequent detection of subclinical coronary plaques in SLE patients, particularly among those with renal impairment and lupus nephritis, underscoring the interplay between systemic inflammation, organ damage, and vascular remodeling [91]. Stojan et al. further demonstrate that high-risk plaque characteristics are more prevalent in SLE and are associated with disease activity, suggesting that immune activation may influence plaque phenotype and vulnerability rather than merely plaque burden [92].

Moreover, structural plaque assessment does not fully capture the spectrum of coronary involvement in SLE [93]. In a combined PET-CT study, Weber et al. show reduced myocardial flow reserve and a high prevalence of coronary microvascular dysfunction in SLE patients presenting with chest pain, despite similar epicardial plaque burden compared with controls [93]. These findings indicate that coronary pathology in SLE extends beyond macrovascular stenosis and includes functional microvascular impairment [93].

Collectively, cCT-based studies demonstrate that SLE is associated with early and multifaceted coronary involvement, including accelerated calcification, subclinical non-obstructive plaques, high-risk plaque features, and microvascular dysfunction (Table 2). These observations support the integration of cCT techniques into CV risk stratification algorithms for selected high-risk SLE patients. In this context, coronary CT angiography (CCTA) may play a pivotal role in identifying subclinical coronary atherosclerosis and refining CV risk stratification, thereby supporting the early implementation of preventive strategies, including lipid-lowering therapies in high-risk individuals.

### 5.3. CMR Assessment in SLE Patients

CMR has emerged as the reference imaging modality for the comprehensive assessment of myocardial involvement in SLE, owing to its ability to combine functional evaluation with detailed tissue characterization [95,96] (Table 3). Early studies demonstrate a high prevalence of subclinical immune-mediated myocardial inflammation in SLE, frequently detected by early gadolinium enhancement and T2-weighted imaging, even in the absence of significant LGE, supporting an inflammatory rather than viral aetiology [95].

Multiparametric approaches including native T1 mapping and extracellular volume (ECV) quantification may reveal diffuse myocardial abnormalities in SLE patients without CV disease [97]. Elevated native T1 and ECV values, consistent with diffuse interstitial fibrosis, have been reported even in patients with preserved ejection fraction, underscoring the presence of silent myocardial remodeling [97]. In patients presenting with HF, CMR allows aetiologic differentiation between active myocarditis, ischemic injury, vasculitis-related damage, and cardiomyopathy, with the extent of LGE correlating with disease activity [98].

Quantitative techniques may further refine the detection of early myocardial injury. Increased myocardial T2 values in clinically inactive SLE patients indicate diffuse subclinical myocardial oedema despite normal LVEF and absence of focal fibrosis, suggesting ongoing low-grade inflammation [99]. In contrast, LGE-based studies have demonstrated a significant prevalence of non-ischemic mid-wall fibrosis, often associated with diastolic dysfunction and reduced exercise capacity, highlighting the transition from inflammation to chronic fibrotic remodeling [100].

Importantly, CMR may detect occult myocardial involvement in symptomatic SLE patients with normal TTE, revealing silent myocarditis, infarction, or vasculitic subendocardial fibrosis that would otherwise remain undiagnosed [101]. In newly diagnosed, treatment-naïve patients, elevated native T1 and ECV values have been documented even before the appearance of LGE or functional impairment, suggesting that quantitative CMR markers identify the earliest stages of immune-mediated myocardial injury [102].

Longitudinal data indicate that subclinical myocarditis detected by CMR does not necessarily progress to overt clinical myocarditis over short-term follow-up, although tissue abnormalities may persist despite functional improvement and intensified immunosuppression [103]. More recently, associations between focal myocardial fibrosis and LAC positivity have been described, supporting a possible link between APS-related microvascular injury and myocardial remodeling [96].

In this context, the identification of myocardial scar and viability assumes additional clinical relevance. Although traditionally investigated in CAD, the assessment of myocardial viability using advanced imaging techniques may also be informative in SLE, where microvascular dysfunction and immune-mediated injury can lead to ischemic-like myocardial damage. CMR, through LGE and mapping techniques, enables accurate characterization of myocardial scar and differentiation between viable and non-viable tissue, thereby contributing to a more comprehensive interpretation of LV dysfunction in this population [104].

Collectively, these findings position CMR as a highly sensitive modality for detecting both inflammatory and fibrotic myocardial involvement in SLE, offering incremental diagnostic and pathophysiological insights beyond conventional TTE and enabling refined CV risk stratification. However, it should be acknowledged that most of the available evidence is derived from relatively small, single-center, observational studies, and therefore these findings should be interpreted with caution.

Given the heterogeneity of CV manifestations in SLE, no single imaging modality is sufficient to capture the full spectrum of myocardial and vascular involvement. A structured comparison of the diagnostic capabilities, advantages, and limitations of each modality is provided in Table 4, supporting a multimodal and risk-adapted imaging strategy.

## 6. Imaging as a Tool for Guidance and Prevention

### 6.1. Multimodality Imaging as a Valuable Tool in Guiding CV Prevention

Beyond its diagnostic role, multimodal cardiac imaging represents a pivotal instrument for guiding CV prevention in SLE [104]. By detecting subclinical myocardial inflammation, diffuse fibrosis, atrial remodeling, coronary microvascular dysfunction, and early atherosclerotic changes, imaging allows identification of high-risk phenotypes long before CV events occur [105]. This early recognition enables timely optimization of aggressive management of traditional and SLE-related risk factors, and implementation of tailored cardioprotective strategies [105]. Moreover, serial imaging provides a dynamic assessment of disease activity and therapeutic response, helping distinguish immune-mediated injury from treatment-related cardiotoxicity or conventional CV pathology [105]. In this context, an imaging-guided, risk-adapted approach supports a shift from reactive treatment of established cardiac damage to proactive CV prevention, aligning with the principles of precision medicine in SLE [106].

It is important to recognize that the strength of evidence varies across imaging modalities. TTE remains a well-established first-line tool with broad clinical validation, whereas CMR and cCT provide incremental insights supported by an expanding but predominantly observational evidence base. In contrast, advanced techniques such as deformation imaging and quantitative CMR parameters (including T1, T2 mapping, and extracellular volume) should be considered emerging tools, as current data are largely derived from small, single-center studies and require further validation.

### 6.2. Primary and Secondary Prevention Measurements in Patients with SLE

Given the central role of chronic low-grade inflammation in driving both myocardial remodeling and vascular dysfunction in SLE, preventive strategies should also address modifiable pro-inflammatory factors, including dietary patterns [107]. Growing evidence suggests that pro-inflammatory dietary patterns may contribute to CV risk in SLE. In this context, higher Dietary Inflammatory Index (DII) scores have been associated with worse CV risk markers in women with SLE, supporting the hypothesis that dietary modulation of inflammation may represent a relevant adjunctive strategy for cardiovascular prevention in this population [108].

Adoption of an anti-inflammatory dietary approach, such as the Mediterranean diet, may represent an adjunctive non-pharmacological strategy to mitigate CV risk in this high-risk population [107,109]. Nutritional interventions aimed at reducing systemic inflammation have been increasingly explored in SLE. Recent literature reviews highlight that dietary modulation may influence lipid profiles, oxidative stress, endothelial function, and overall cardiovascular risk in SLE patients [110].

In this framework, CV management in patients with SLE should follow a risk-adapted and stage-specific approach. In patients without overt CV disease, primary prevention strategies should focus on early identification of subclinical involvement through multimodal imaging, aggressive management of traditional CV risk factors, and optimal control of systemic inflammation, as already said [107,108,109,110]. In this setting, imaging modalities such as TTE, CMR and cCT may contribute to reassessing the risk and support timely preventive interventions, including lipid-lowering therapies in selected high-risk individuals.

In contrast, in patients with established CV disease, secondary prevention should be guided by current guideline-based recommendations, including antiplatelet therapy, statins, blood pressure control, and, when appropriate, revascularization strategies [111]. In addition to lifestyle and risk factor modification, anti-inflammatory pharmacological strategies have emerged as a potential adjunctive approach for CV prevention. In this context, colchicine has gained increasing attention due to its ability to inhibit key inflammatory pathways involved in atherosclerosis, including the NOD-like receptor family pyrin domain containing 3 (NLRP3) inflammasome and interleukin-1 signalling [112]. Large randomized trials in patients with CAD have demonstrated that low-dose colchicine reduces the risk of major adverse CV events, supporting the concept that targeting residual inflammatory risk may provide incremental benefit beyond traditional therapies [113].

Although specific evidence on SLE is still limited, colchicine may represent a promising adjunctive strategy in selected high-risk patients. However, dedicated studies are needed to clarify its efficacy and safety in this population, as well as its interaction with immunosuppressive therapies.

**Figure 6:**
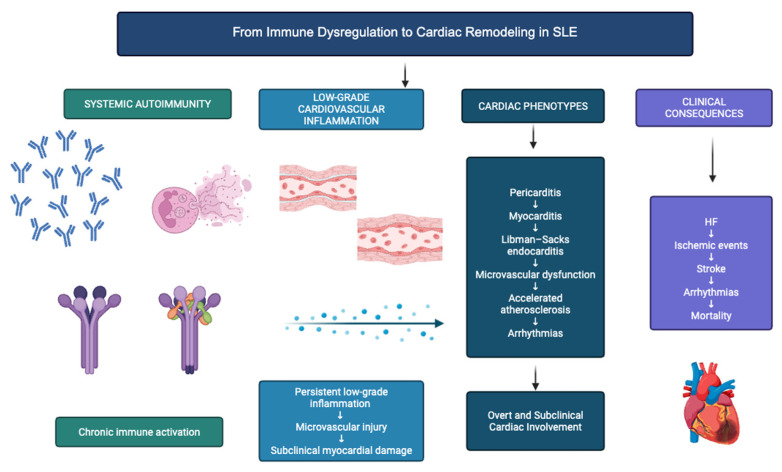
From immune dysregulation to cardiac remodeling in systemic lupus erythematosus.

Overall, the integration of multimodal imaging into cardiovascular management pathways may facilitate a transition from reactive to proactive and personalized care in patients with SLE [114] (Figure 6).

### 6.3. Gaps in Knowledge

Despite significant progress in multimodal cardiac imaging, important gaps remain in the cardiovascular management of SLE.

Quantitative imaging biomarkers—such as global longitudinal strain, left atrial strain, native T1 mapping, extracellular volume, and T2 mapping—have shown high sensitivity for detecting subclinical myocardial involvement. However, lack of standardized cut-offs and limited prospective outcome validation currently restrict their integration into routine risk stratification. Hybrid techniques, including PET–CMR, may enable simultaneous assessment of inflammatory activity and structural remodelling, facilitating differentiation between active immune-mediated injury and chronic fibrosis. This distinction could prove relevant for guiding immunomodulatory therapy and monitoring response. Artificial intelligence and automated image analysis represent additional promising directions helping identify high-risk cardiovascular phenotypes in SLE.

Future prospective studies should define optimal screening strategies, validate imaging biomarkers against hard cardiovascular outcomes, and clarify their role as surrogate endpoints in interventional trials. Advancing these areas will be essential to fully integrate multimodal imaging into precision cardio-rheumatology.

## 7. Conclusions

CV involvement in SLE represents a multifaceted and dynamic process driven by the interplay between chronic immune activation, low-grade inflammation, microvascular dysfunction, and accelerated atherosclerosis [1]. Increasing evidence demonstrates that myocardial and vascular injury often precede clinical manifestations, remaining silent yet biologically active for years [5]. In this context, multimodal cardiac imaging has transformed our understanding of SLE-related CV disease [105]. Beyond its diagnostic utility, imaging plays a pivotal role in risk stratification and clinical decision-making. Importantly, chronic low-grade inflammation emerges as a unifying pathophysiological mechanism linking myocardial remodeling and vascular disease in SLE [105]. Therefore, CV prevention should not rely solely on pharmacologic strategies but also incorporate modulation of inflammatory burden through lifestyle interventions [106]. Attention to anti-inflammatory dietary patterns, such as the Mediterranean diet, alongside physical activity and weight control, may represent complementary tools in long-term CV protection [106,107].

In conclusion, a comprehensive, imaging-informed—including TTE, CMR, cCT, and nuclear imaging techniques—and prevention-oriented strategy is essential to shift the paradigm from reactive treatment of established cardiac damage to proactive CV protection [1]. Integrating advanced imaging with personalized risk modification embodies the principles of precision medicine and offers a promising path toward reducing CV morbidity and mortality in SLE. However, despite growing evidence, important limitations remain, including the lack of standardized imaging thresholds, limited prospective validation, and heterogeneity of study designs.

Future studies are needed to validate imaging biomarkers, define their prognostic value, and establish their role in guiding therapeutic decision-making.

## Figures and Tables

**Table 1:** TTE assessment of cardiac involvement in SLE (studies of last 10 years).

Study	Population	TTE Approach	Main Cardiac Findings	Clinical Relevance
Zhong et al., 2025 [87]	145 patients with SLE and 57 controls	TTE with GLS assessment and assessment of LA mechanics	Increased LA stiffness and impaired reservoir function, particularly in patients with SLE- nephritis.	LAS index can be used as early detector of subclinical DD
Shahab et al., 2025 [83]	55 patients with SLE and 31 controls	TTE with GLS assessing LV deformation	Increased mechanical dispersion despite preserved ejection fraction; complement protein C4 predicts LVMD	Identification of early DD and inflammatory burden
Morello et al., 2025 [76]	76 patients with SLE	TTE with GLS assessment	Reduced GLS in 24% of patients; associated with DD, RV impairment, pericardial involvement, and elevated inflammatory markers	Subclinical systolic dysfunction is linked to cumulative disease activity and immune-mediated myocardial injury
Kadoglou et al., 2024 [77]	82 patients with SLE and 41 controls	Arterial stiffness assessment, blood analysis and GLS	Impaired myocardial strain and arterial stiffness assessment are typical of SLE patients	Combined cardiac–vascular evaluation improves CV risk stratification in SLE patients
Lai et al., 2024 [78]	286 patients with SLE and 100 matched controls	TTE with GLS assessing LV deformation	Increased LV mass and wall thickness, impaired DD, and altered myocardial performance indices; abnormalities correlated with disease activity and CV events	Doppler TTE enables early detection of DD and identifies patients at higher risk of future CV events
Myhr et al., 2022 [79]	108 patients with SLE (longitudinal follow-up)	TTE	Progressive impairment of DD and LA enlargement over time in patients with LAC	Evidence of gradual cardiac remodeling in patients with LAC
Mohamed et al., 2019 [80]	59 patients with SLE	TTE	High prevalence of subclinical valvular abnormalities and pericardial effusion in SLE patients	TTE features identify clinical predictors of SLE progression
Sun et al., 2018 [81]	102 patients with SLE stratified by severity of PH and 30 controls	TTE with GLS assessing RA function	Progressive enlargement of RA volumes and increased active emptying with worsening PH; reduced passive function and systolic strain parameters	Speckle-tracking analysis detects RA functional remodeling according to PH severity
Dai et al., 2016 [82]	60 patients with SLE and matched controls	TTE with GLS assessment, LA strain and strain rate	Reduced LA reservoir and conduit function, increased pump function, and enlarged LA volume; abnormalities associated with severity of DD and cumulative disease damage	TTE identifies predictors of SLE pathologies

Abbreviations: CV: cardiovascular; DD: diastolic dysfunction; GLS: global longitudinal strain; LA: left atrium; LAC: lupus anticoagulant antibodies; LV: left ventricle; LVMD: left ventricle myocardial dispersion; PH: pulmonary hypertension; RA: right atrium; RV: right ventricle; SLE: systemic lupus erythematosus; TTE: trans-thoracic echocardiography.

**Table 2:** cCT assessment in SLE patients.

Study (Year)	Population	CT Technique	Main Findings	Clinical Implications
Yiu et al., 2009 [88]	Patients with SLE without known CAD	Electron-beam cCT	Higher prevalence of CAC in SLE patients compared with controls (in coronary arteries, carotids and aorta); earlier onset of calcified plaque	Evidence of premature atherosclerosis in SLE
Mavrogeni et al., 2017 [44]	SLE patients	cCT. CMR, TTE and laboratory tests	Identification of subclinical coronary atherosclerosis	Useful for early risk stratification
Khan et al., 2017 [89]	36 SLE- patients vs. matched controls	Coronary calcium scoring and plaque evaluation	Increased CAC in SLE- patients independent of traditional and SLE-related CV risk factors	Suggests SLE-related mechanisms promote vascular calcification
Hermansen et al., 2018 [90]	147 SLE patients	cCT	Non-obstructive coronary plaques frequently detected even in asymptomatic patients (especially with impaired renal function and lupus nephritis)	Supports early coronary imaging in selected high-risk patients
Gartshteyn et al., 2019 [91]	76 SLE patients and controls	Non-contrast CT calcium scoring	CAC scoring useful for early detection of plaque	Supports CAC scoring for CV risk stratification in SLE
Stojan et al., 2020 [92]	72 SLE patients and controls	cCT	Association between high-risk plaque characteristics and disease activity in SLE patients compared to controls	Disease activity may influence plaque phenotype
Weber et al., 2021 [93]	42 SLE patients with chest pain (no obstructive coronary disease) vs. matched controls	PET–cCT	Reduced myocardial flow reserve and high prevalence of coronary microvascular dysfunction in SLE patients despite similar plaque burden	Demonstrates microvascular disease independent of macrovascular stenosis
Wu et al., 2023 [94]	SLE patients in primary prevention setting	Non-contrast CT calcium scoring	CAC scoring useful for early detection of plaque burden; zero score strong negative predictor	Supports CAC scoring for CV risk stratification in SLE

Abbreviations: CAC: coronary artery calcification; cCT: cardiac computed tomography; CAD: coronary artery disease; PET: positron emission tomography; SLE: systemic lupus erythematosus.

**Table 3:** CMR assessment in SLE patients.

Study (Year)	Population	CMR Technique	Main Findings	Clinical Implications
Mavrogeni et al., 2013 [95]	Patients with suspected myocarditis (20 SLE patients and 20 controls)	CMR with T2-weighted imaging, EGE and LGE; biopsy correlation	High prevalence of subclinical myocardial inflammation in SLE; EGE frequent, limited LGE; viral genome typically absents in SLE	CMR detects immune-mediated myocardial involvement in SLE and aids differentiation from viral myocarditis, supporting early immunomodulatory management
Puntmann et al., 2013 [97]	SLE patients without known CV disease	Multiparametric CMR including native T1 mapping, ECV quantification and LGE	Elevated native T1 and ECV indicating diffuse myocardial fibrosis; LGE present in 61% despite preserved ejection fraction	Demonstrates subclinical diffuse myocardial involvement in SLE and highlights T1 mapping as a sensitive early marker of immune-mediated myocardial remodeling
Mavrogeni et al., 2014 [98]	32 patients with SLE and recent-onset HF	CMR with T2-weighted imaging and LGE	Distinct etiologic patterns identified (active myocarditis, dilated cardiomyopathy, myocardial infarction, vasculitis, valvular disease); extent of LGE correlated with disease activity and duration	CMR enables etiologic differentiation of HF in SLE and detects inflammatory and ischemic myocardial injury with prognostic implications
Zhang et al., 2015 [99]	24 patients with SLE with low disease activity and 12 healthy controls	CMR with quantitative T2 mapping, native T1 mapping, cine imaging and LGE	Significantly increased myocardial T2 values in SLE despite preserved ventricular function and absence of LGE, indicating diffuse subclinical myocardial oedema	Quantitative T2 mapping detects low-grade immune-mediated myocardial inflammation in clinically inactive SLE
Seneviratne et al., 2016 [100]	41 patients with SLE without overt HF	CMR with LGE and functional assessment	Myocardial fibrosis detected in 37% of patients, predominantly with a non-ischemic mid-wall pattern; greater fibrosis associated with diastolic impairment and reduced exercise capacity	CMR identifies silent inflammatory myocardial fibrosis in SLE
Mavrogeni et al., 2018 [101]	80 SLE patients with atypical cardiac symptoms and normal TTE	Cine imaging, T2-weighted imaging, LGE)	Occult myocardial involvement detected in 27.5% (silent myocarditis, silent myocardial infarction, diffuse subendocardial fibrosis due to vasculitis) despite normal EF	CMR identifies subclinical cardiac lesions missed by TTE
Guo et al., 2018 [102]	50 newly diagnosed treatment-naïve SLE patients, 60 long-standing SLE patients, and 50 healthy controls	Native T1 mapping, ECV quantification, and LGE	Elevated native T1 and ECV detected in newly diagnosed SLE patients despite normal EF and absence of LGE; more advanced disease showed fibrosis	CMR identifies early diffuse myocardial involvement in SLE before functional decline
du Toit et al., 2020 [103]	Hospitalized patients with SLE undergoing longitudinal follow-up (12 months)	CMR using Lake Louise criteria (T2-weighted imaging, early and LGE) with parallel TTE assessment	Subclinical myocarditis frequently detected but does not progress to clinical myocarditis over 12 months; myocardial oedema and LGE often persisted despite functional improvement	CMR identifies persistent subclinical myocardial inflammation in SLE; however, short-term prognostic impact appears limited
Myhr et al., 2024 [96]	79 patients with SLE and 79 matched controls	Native T1 mapping, ECV quantification, and LGE	Increased native T1 values and focal myocardial fibrosis detected in SLE; LGE independently associated with LAC positivity	Links antiphospholipid profile to myocardial injury

Abbreviations: CMR: cardiac magnetic resonance; CV: cardiovascular; ECV: extracellular volume; EF: ejection fraction; EGE: early gadolinium enhancement; HF: heart failure; LAC: lupus anticoagulant; LGE: late gadolinium enhancement; SLE: systemic lupus erythematosus; TTE: trans-thoracic echocardiography.

**Table 4:** Comparative overview of the principal cardiac imaging modalities and limitations in systemic lupus erythematosus. This comparative framework supports a multimodal, patient-tailored imaging strategy.

Imaging Modality	Main Cardiac Domains	Early Inflammation Detection	Fibrosis Detection	Coronary Atherosclerosis Assessment	Microvascular Dysfunction Assessment	Radiation Exposure	Strengths	Limitations
TTE (including speckle tracking)	Ventricular function, valvular disease, pericardial effusion, atrial remodeling	Indirect (via strain abnormalities)	No direct tissue characterization	No	Indirect (diastolic indices, strain)	No	Widely available; low cost; bedside applicability; suitable for serial follow-up	Operator-dependent; limited tissue characterization; lower sensitivity for early myocardial inflammation
CMR	Myocarditis, fibrosis, edema, ventricular volumes, pericardial inflammation	Yes (T2 mapping, early gadolinium enhancement)	Yes (LGE, native T1 mapping, ECV quantification)	Limited (not primary modality for coronary lumen assessment)	Indirect	No	Gold standard for myocardial tissue characterization; detects subclinical involvement	Higher cost; limited availability; contraindications in some patients; contrast limitations in renal dysfunction
Cardiac CT (Calcium scoring)	Coronary calcification, plaque burden, high-risk plaque features, pericardial anatomy	No	No	Yes (excellent anatomical assessment)	No	Yes	High spatial resolution; strong negative predictive value of zero calcium score	Radiation exposure; contrast nephrotoxicity risk; limited functional information
PET (± CT)	Vascular inflammation, myocardial perfusion	Yes (metabolic activity assessment)	No	Indirect (inflammatory plaque activity)	Yes (myocardial flow reserve)	Yes	Functional assessment of inflammation and microvascular dysfunction	High cost; radiation exposure; limited availability
Hybrid PET–CMR	Combined structural and metabolic myocardial assessment	Yes	Yes	Limited	Yes	Variable	Comprehensive inflammation–fibrosis–perfusion evaluation	Mainly research setting; limited accessibility

Abbreviations: CMR: cardiac magnetic resonance; CT: computed tomography; ECV: extracellular volume; LGE: late gadolinium enhancement; PET: positron emission tomography; TTE: trans-thoracic echocardiography.

## Data Availability

No new data were created or analyzed in this study.

## Abbreviations

The following abbreviations are used in this manuscript:

Anti Ro-SSA	anti-Sjögren’s-syndrome-related antigen A
APS	anti-phospholipid syndrome
CAC	coronary artery calcification
cCT	cardiac computed tomography
CMR	cardiac magnetic resonance
CV	cardiovascular
ECV	extracellular volume
GLS	global longitudinal strain
HF	heart failure
LA	left atrial
LGE	late gadolinium enhancement
LN	lupus nephritis
LVEF	left ventricular ejection fraction
LVMD	left ventricle mechanical dispersion
NET	neutrophil extracellular trap
NLRP-3	NOD-like receptor family pyrin domain containing 3
OxLDL	oxidized low- density lipoprotein
PET	positron emission tomography
PH	pulmonary hypertension
SLE	systemic lupus erythematosus
TTE	trans-thoracic echocardiography
